# The Impact of *BRAF* Mutation Status on Survival Outcomes and Treatment Patterns among Metastatic Colorectal Cancer Patients in Alberta, Canada

**DOI:** 10.3390/cancers15245748

**Published:** 2023-12-08

**Authors:** R. Liam Sutherland, Devon J. Boyne, Darren R. Brenner, Winson Y. Cheung

**Affiliations:** 1 Department of Community Health Sciences, University of Calgary, Calgary, AB T2N 4N1, Canada; 2 Department of Oncology, University of Calgary, Calgary, AB T2N 4N1, Canada; 3 Department of Medicine, University of Calgary, Calgary, AB T2N 4N1, Canada

**Keywords:** colorectal cancer, *BRAF*, treatment patterns, survival, real-world evidence, mutation, prognosis

## Abstract

**Simple Summary:**

Colorectal cancer can present in diverse ways due to genetic and molecular variations. This study focused on metastatic colorectal cancer (mCRC) and its correlation with a specific genetic mutation known as v-raf murine sarcoma viral oncogene homolog B1 (*BRAF*). Among the 488 mCRC patients studied, 42 (11.4%) were found to have the *BRAF* mutation. The initial treatment for most patients involved capecitabine and oxaliplatin (CAPOX) or leucovorin calcium (folinic acid), fluorouracil, and oxaliplatin (FOLFOX). The median overall survival for all mCRC patients was approximately 20.01 months. However, those with the *BRAF* mutation experienced significantly poorer outcomes, with a median survival of only 8.21 months compared to 20.03 months for those without the mutation. This study underscores the significance of early *BRAF* testing at the time of colorectal cancer diagnosis. Such testing can help determine a patient’s prognosis and enable the development of personalized treatment strategies, ultimately leading to improved outcomes for individuals with advanced colorectal cancer.

**Abstract:**

Colorectal cancer presents via multiple different clinical phenotypes that can arise from a variety of different genetic and molecular alterations. The aim of this study was to describe survival outcomes and treatment patterns of metastatic colorectal cancer (mCRC) patients by v-raf murine sarcoma viral oncogene homolog B1 (*BRAF*) mutation status. The Alberta Cancer Registry was used to identify all patients >18 years old who had been diagnosed with mCRC in Alberta between 1 January 2017 and 31 December 2019 and had received at least one cycle of systemic therapy. Treatment patterns were compared between wild-type and mutant *BRAF* mCRC patients. Cox regression models and Kaplan–Meier curves were created to assess survival differences by both treatment pattern and *BRAF* status. A total of 488 patients were identified with mCRC, of which 42 (11.4%) were confirmed to have a *BRAF* mutation. The most common first-line treatment regimen was either capecitabine and oxaliplatin (CAPOX) or leucovorin calcium (folinic acid), fluorouracil, and oxaliplatin (FOLFOX). The median overall survival for mCRC patients was 20.01 months. Mutant *BRAF* patients had a median survival of 8.21 months compared to 20.03 months among those with wild-type *BRAF*. *BRAF* mutations among mCRC patients are associated with a considerably poor prognosis, reinforcing the need for clinical *BRAF* testing among newly diagnosed patients to better understand their prognosis.

## 1. Introduction

Colorectal cancer (CRC) is the second most common cancer, accounting for 13% of all incident cancers in Canada [1]. Most often, colorectal adenoma development follows a chromosomal instability (CIN) pathway, resulting in widespread loss of heterozygosity, and gross chromosomal abnormalities [2,3]. Chromosomal alternations often involve a key mutation in the adenomatous polyposis coli (*APC*) tumor suppressor gene [4]. Analysis of the epigenome has shown that almost all cases of CRC have aberrantly methylated genes; a subset of these events was shown to directly contribute to CRC progression [5]. Although a large majority of CRCs develop via an adenomatous pathway, it has been estimated that 20 to 35% of CRC cases arise along the serrated pathway via precursor lesions known as sessile serrated adenomas (SSA) [6,7,8,9,10]. These lesions are believed to arise from aberrant cytosine–phosphate–guanine (CpG) island methylation and are often associated with interval cancers, where the cancer is diagnosed shortly after a negative colonoscopy [11,12]. 

The v-raf murine sarcoma viral oncogene homolog B1 (*BRAF*) gene encodes a serine/threonine protein kinase that is a downstream target of the Kirsten rat sarcoma viral oncogene (*KRAS*) and activates the mitogen-activated protein kinase (MAPK) pathway and is known to be involved with intracellular signaling and cell growth [13]. There are many different types of *BRAF* mutations, but the most common—and the subject of this study—is the *BRAF V600E* mutation, which indicates that a valine was replaced by glutamic acid at amino acid number 600 inside the protein. SSAs are typically the result of *BRAF* mutation or CpG island hypermethylation. Observational studies suggest that 5 to 10% of metastatic CRC cases possess a *BRAF* mutation [14]. Using the large genomic database cBioportal, over 14,000 CRC tumors were tested, and roughly 9% were found to have a *BRAF* mutation [15]. There is evidence that *BRAF* mutations confer uncontrolled growth and proliferation among colorectal carcinoma cells [16]. In addition, *BRAF*-mutated cases typically have a poor prognosis and lower rates of progression-free survival [14,17,18,19]. Survival analyses of patients with early-stage (stages I to III) CRC showed that patients with a *BRAF*-mutated cancer had a decreased overall survival compared to those without the mutation [19]. Further, progression-free survival among metastatic CRC (mCRC) patients has also been shown to be significantly worse for patients possessing *BRAF*-mutated CRC [14]. This is consistent with a growing body of evidence regarding the prognostic and predictive role of *BRAF* mutations across many different cancers, including malignancies of the gastrointestinal tract, lung, and skin (melanoma) [20]. For CRC patients, those with tumors that are *BRAF* mutated and microsatellite stable appear to experience the worse prognosis [20]. Similar observations have been made for melanoma and lung cancer [21]. For both, *BRAF* mutations are more commonly seen in younger patients and are more likely to be associated more aggressive tumor biology [21]. Emerging data suggest that more tailored or targeted treatments for individuals with *BRAF*-mutated tumors may improve outcomes [22].

For these reasons, this study first examined the *BRAF* mutational testing landscape in Alberta, Canada. Through the use both administrative data and chart review, treatment patterns were then characterized and compared between *BRAF*-mutant and wild-type patients. Detailed survival analyses among mCRC patients were performed. Thus, this study aimed to generate real-world evidence to supplement the growing body of research surrounding *BRAF* mutations in the clinical management of colorectal cancer.

## 2. Materials and Methods

### 2.1. Patient Selection and Data Handling

The Alberta Cancer Registry collects and registers clinical information for all cancer patients diagnosed within the province. The registry was used to identify all patients aged ≥18 years with a colon, rectum, or colorectal cancer diagnosis according to International Classification of Diseases codes over a three-year period from 1 January 2017 to 31 December 2019. The registry stages all cancers according to the standards provided by the American Joint Committee on Cancer and based on their coding; all cases of CRC stage IV (IV, IVA, IVB, IVC, & IV NOS) were included in this study. In the case of multiple primary tumors, the tumor with the highest stage was included. The resulting cases then underwent a thorough chart review by medically trained abstractors to obtain the detailed information surrounding *BRAF* testing. Small cell sizes (<10 patients) were censored to ensure the data remained non-identifiable. Data pertaining to all patients’ treatment were obtained through the Pharmacy Information Network, Discharge Abstract Database, and the National Ambulatory Care Reporting System databases, which were merged with the Alberta Cancer Registry. 

### 2.2. Statistical Analyses

#### 2.2.1. Patient Population

Patient baseline demographics (age, sex, year of diagnosis, cancer site, and sites of metastasis) were summarized. Mean age and standard deviation were calculated for all patients. Information regarding sex, year of diagnosis, primary cancer site, and site of metastases was provided. Differences were examined for *BRAF* status (mutant, wild type, not tested), and statistical significance was determined via a two-tailed *t*-test or a Chi-squared test, where a resulting *p*-value of <0.05 was deemed significant. Only patients who started systemic therapy were included in the survival analyses.

#### 2.2.2. *BRAF* Test Characteristics

Prevalence of *BRAF* testing was determined using a simple proportion of those who were tested for *BRAF* mutation compared to the total number of patients identified with mCRC from the administrative data. Timing of testing was estimated by taking the number of days from the date the test was ordered to the date the test results were received. Summary statistics including quartile and median were calculated. Other information such as testing location, testing year, type of test used, and specialty of ordering physician was reported as count data. 

#### 2.2.3. Treatment Patterns

Treatment information was organized into lines of systemic therapy based on associated claims data and start and end date of the specific therapy. Based on the agents administered, specific regimens were defined. CAPOX or FOLFOX was defined if patients received either Capecitabine (Xeloda) and Oxaliplatin (Eloxatin) or Fluorouracil (5-Fluorouracil, Efudex, Fluoroplex) and Leucovorin Calcium and Oxaliplatin (Eloxatin). FOLFIRI was defined if a patient was given Fluorouracil (5-Fluorouracil, Efudex, Fluoroplex) and Irinotecan and Leucovorin Calcium. Capecitabine was defined if a patient was administered Capecitabine (Xeloda). FOLFIRI + monoclonal antibody (MAB) and CAPOX or FOLFOX + MAB were defined if a patient was administered with either the FOLFIRI or CAPOX + FOLFOX regimen and a monoclonal antibody therapy. Finally, any other agents outside of the predefined regimens were classified as Other. From this point, the first-, second-, third-, and fourth-line regimens were summarized and compared between *BRAF* statuses. Duration of therapy was defined as the number of days between the date the regimen was first administered to the date of last administration. Also, the time to next treatment was estimated as the date from first administration to the date of first administration of the subsequent line. The first and third quartile and the median number of days of therapy was calculated. Sequencing of treatment lines was also determined to understand the specific treatment patterns used within this population and if it differed by *BRAF* status. Comparisons were made using a two-tailed *t*-test with a *p*-value <0.05 deemed significant.

#### 2.2.4. Survival Analyses 

Overall survival (OS) was defined using the date of diagnosis as the start point for the overall analyses. The treatment line start date was used as the start point for the treatment regimen subgroup analysis. Median survival in months (95% confidence interval (CI)) and 1- and 2-year survival probabilities (95% CI) were also calculated for all analyses.

Cox regression models were used to estimate survival outcomes, and models were stratified by *BRAF* mutation status and treatment group. Similarly, Kaplan–Meier curves were produced stratified by *BRAF* mutation status and treatment group. OS was assessed for all patients, and then based on treatment regimen and *BRAF* status using both all-cause mortality and cancer-specific mortality, and for the *BRAF* status subgroup analyses.

## 3. Results

### 3.1. Sample Characteristics and BRAF Testing

Baseline characteristics and *BRAF* testing information are summarized in Table 1. The sample consisted of 488 patients who were diagnosed with mCRC between January 2017 and December 2019. The mean age of the sample was 63.3 years, and roughly 60.4% of patients were male. Most patients were diagnosed in 2017 (46.7%), with 29.1% being diagnosed in 2018 and 24.2% being diagnosed in 2019. In total, 71.5% of cases had primary tumors in the colon, with 25.2% being in the rectum and 3.3% at the rectosigmoid junction. A large majority of cases presented with metastases to the liver (69.9%), and 28.1% and 25.4% had metastases to the lungs and lymph nodes, respectively. Approximately three in four patients underwent *BRAF* testing, and, of those tested, an estimated 11.4% were shown to be *BRAF* mutant. The median number of days to receive test results was 11.5 (interquartile range (IQR): 8–25). 

### 3.2. Treatment Patterns

Treatment patterns were assessed for all patients that initiated systemic therapy based on administrative claims data. In total, 327 (67.0%) initiated a first-line therapy, and 194 (39.8%) initiated a second-line therapy (Table 2). There were no significant differences when comparing the treatment regimens received between *BRAF*-wild-type and -mutant patients. The most common first-line therapy regimen was CAPOX or FOLFOX at 36.4%, and 22.9% of patients received a Capecitabine regimen as their first-line therapy. Overall, 194 (59.3%) patients were also shown to have initiated second-line therapy, where 27.8% of patients received a CAPOX or FOLFOX regimen, and 25.3% of patients received a FOLFIRI regimen. The duration for first-through fourth-line therapy is summarized in Table 3. It took a median of 168 days (IQR: 84.5–244.5) from initiation to end of first-line therapy and 101.5 days (IQR 63–196) from initiation to end of second-line therapy. The median number of days from the initiation of first-line therapy to the initiation of second-line therapy was 246 days (IQR: 171–367.8). Information for third- and fourth-line treatment patterns can be found in Supplementary Table S1.

### 3.3. Survival Analyses

**Figure 1:**
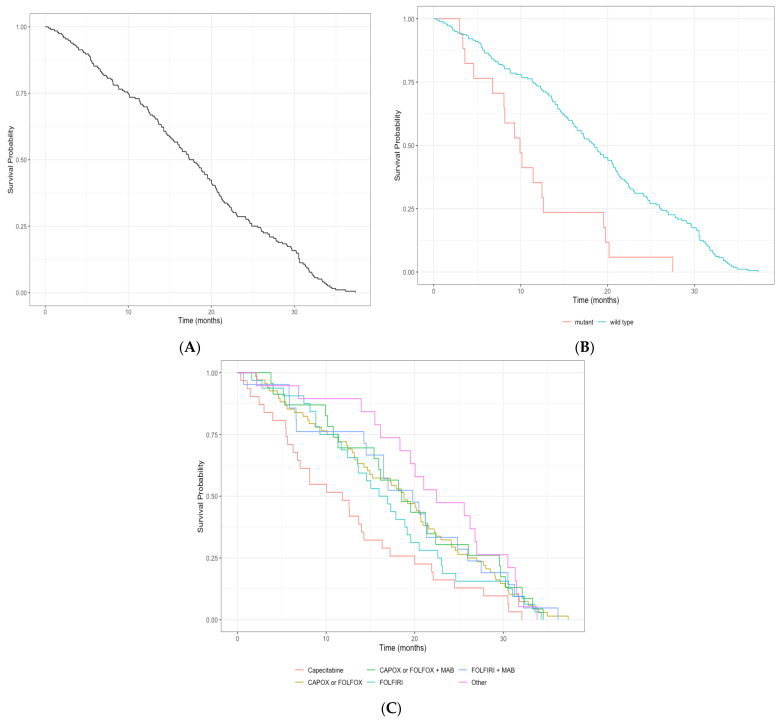
Kaplan–Meier curves presenting survival from time of initiation of first-line therapy to all-cause death among metastatic colorectal patients in Alberta. (**A**) Overall survival (**B**) stratified by *BRAF* status, (**C**) stratified by first-line therapy regimen.

OS from initiation of first-line therapy to all-cause mortality and OS from initiation of first-line therapy to cancer-specific mortality are summarized in Table 4 and Table 5, respectively. Further, Kaplan–Meier curves for OS from time of first-line therapy, survival stratified by *BRAF* status, and survival stratified by treatment regimen can be found in Figure 1A, 1B, and 1C, respectively. Median survival from initiation of first-line therapy to all-cause mortality was 20.01 months (95% CI: 15.97–22.21), with 1- and 2-year survival probabilities of 0.66 (95% CI: 0.61–0.72) and 0.38 (95% CI: 0.32–0.44), respectively. Among patients with a wild-type *BRAF*, median survival from initiation of first-line therapy to all-cause mortality was 20.03 months (95% CI: 17.87–22.83), with 1- and 2-year survival probabilities of 0.71 (95% CI: 0.65–0.77) and 0.39 (95% CI: 0.21–0.47), respectively. Those patients with a *BRAF* mutation had a significantly worse median survival at 8.21 months (95% CI: 6.54–12.62). Similarly, 1- and 2-year survival probabilities were also significantly worse at 0.30 (95% CI: 0.16–0.56) and 0.16 (95% CI: 0.06–0.43). Since a large majority of mCRC patients eventually die from their disease, many of the results were similar when looking specifically at cancer-specific mortality. The median OS in months from initiation of first-line therapy to cancer-specific mortality was 22.34 months (95% CI: 19.74–24.38), and 1- and 2-year survival probabilities were 0.71 (95% CI: 0.66–0.77) and 0.45 (95% CI: 0.39–0.52). Survival analyses for overall and cancer-specific survival can be found in Supplementary Tables S2 and S3 and Supplementary Figure S1. Results for third- and fourth-line therapies were largely censored due to small cell sizes and thus were not included.

## 4. Discussion

This study is one of the first to characterize the *BRAF* testing landscape in Alberta, Canada, and to further understand how *BRAF* status affects treatment patterns and survival among mCRC patients. Using the Alberta Cancer Registry and retrospective chart review, it was determined that roughly three in four CRC patients currently undergo *BRAF* testing. Further, it takes a roughly two weeks to receive test results from date of order. When classifying treatment patterns among mCRC patients, only two thirds initiated systemic therapy, and the most common first-line therapies were CAPOX and FOLFOX. Treatment patterns were unaffected by *BRAF* status. On average, patients who initiated first-line therapy survived for roughly two years, whereas those with *BRAF* mutations had a significantly shorter survival time. Given that most mCRC patients will eventually succumb to their disease, cancer-specific survival results did not differ markedly. The findings of this study support the continued use of *BRAF* testing for prognostication and treatment decisions.

The current body of research regarding *BRAF* testing among CRC patients is limited. First, this study reported a *BRAF* mutation prevalence of roughly 11%, congruent to the study from Vaughn et al., which reported a prevalence of 15%; however, the study was limited to mCRC patients, and thus this could account for the slightly lower prevalence [23]. Regarding treatment patterns, a recent study from Canada also showed that CAPOX and FOLFOX treatment regimens were most common among CRC patients, likely due to their noted high tolerability and completion rates [24]. Further, our analyses suggest that *BRAF*-wild-type mCRC patients had a median survival of roughly 24 months, which aligns with previous studies among mCRC patients that showed an OS among *BRAF* wild type of roughly 30 months [25,26]. Similarly, the results indicated a 1-year survival probability of 0.71 for *BRAF* wild type, where current studies show roughly an 0.80 1-year survival probability [25,26]. Finally, this study noted a significantly decreased survival for *BRAF*-mutant patients; this is further supported by a previous systematic review and meta-analysis that found an increased mortality from *BRAF* mutation with a hazard ratio (HR) of 2.24 (95% CI, 1.82–2.83) when compared to *BRAF*-wild-type patients [27]. Interestingly, within this review, one study reported a difference in survival between left- and right-sided colon cancer, where a protective HR was reported for left-side colon cancer and a higher HR for right-sided colon cancer [28]. While this study aligns with current literature, it also represents the first study to provide the Canadian perspective on *BRAF* testing and corresponding treatment patterns for mCRC patients. Overall, these results support the continued use of testing for prognostication and treatment decisions. 

Despite the clinical usefulness of *BRAF* testing, still only three in four patients are currently receiving testing. A main barrier that possibly explains why 25% of patients are not tested is likely a result of the lack of research regarding the predictive ability of *BRAF* testing. Our study sample is limited to patients who received *BRAF* testing in 2017, 2018, and 2019. Prior to 2019, research was limited to studies that discerned *BRAF* as simply a negative prognostic marker, and there was not strong evidence to suggest that *BRAF* mutation status could inform treatment decisions through the prediction of treatment response. In 2019, the results of the Binimetinib, encorafenib, and cetuximab (BEACON) trial were published and indicated that patients who received the triple combination displayed improved overall survival and improved median survival [29]. Ultimately, this trial was the first randomized controlled trial to indicate improved survival among *BRAF*-mutant mCRC patients with the use of triple-combination targeted therapies compared to traditional cytotoxics. Further, while there may not currently be any *BRAF*-specific treatment regimes approved for use in Canada, more nuanced treatment decisions such as the decision to perform metastasectomy could be influenced. In 2017, Seligmann and colleagues published a study that indicated the patients with *BRAF*-mutant mCRC are more likely to have a worse prognosis even after metastasectomy [30]. This information could allow clinicians to make more informed decisions as to whether a patient should undergo surgery with advanced-stage disease [30]. It is likely that, as research continues to indicate that *BRAF* status is both prognostic and predictive, *BRAF* testing will continue to increase among mCRC patients. 

This study has several strengths, including, most notably, the quality of data utilized for the analyses. Within Alberta, a cancer diagnosis is a reportable disease, and therefore the Alberta Cancer Registry captures upwards of 99% of CRC cases diagnosed within the province. Therefore, it is likely that this study was able to fully capture the mCRC population, and thus the results can be fully generalizable to future diagnoses both in Alberta and the rest of Canada. Further, patient data within the registry were merged with administrative claims data, and then all included patients underwent a full chart review. Complete and accurate data generated from a single-payer health care system ultimately reduced missing data, ensured accuracy, and reduced potential bias. Despite the strengths, limitations were still present within the current study. First, despite the thoroughness of the Alberta Cancer Registry, it does not capture recurrent cases of cancer, and thus the study relied on an administrative data algorithm to classify recurrent cases. While we have used similar approaches in previous analyses and showed a high sensitivity, this could have led to possible misclassification, though this is expected to be minimal. Further, the administrative data sources do not routinely collect information on potentially important covariates including treatment side effects or toxicity that could provide further information on the treatment pattern landscape for mCRC. Finally, though this study is well equipped to describe the treatment patterns associated with mCRC, it is not designed to evaluate the comparative efficacy or safety of specific treatment regimens between *BRAF*-wild-type or -mutant patients.

## 5. Conclusions

As one of the first studies to describe the treatment patterns for mCRC and the *BRAF* testing landscape in Alberta, Canada, this study indicated that currently three in four patients are routinely tested for *BRAF* mutation and that those who are *BRAF* mutant have a significantly decreased OS. Further, current treatment patterns indicate that a CAPOX or FOLFOX regimen is the most common first-line treatment and that patterns do not differ between *BRAF*-wild-type and -mutant mCRC cases. With the knowledge that *BRAF*-mutant patients survive for significantly shorter amounts of time, these results emphasize the need for both continued testing to further improve prognostication and further research into the need for targeted therapies, specifically *BRAF*-mutant-specific treatment options to improve patient survival and care. 

## Figures and Tables

**Table 1:** Baseline demographics and *BRAF* testing information.

Characteristics	*BRAF* Status				
	Overall	Mutant	Wild Type	Not Tested	*p*
	*n* = 488	*n* = 42	*n* = 325	*n* = 121	
Mean Age (SD)	63.26 (13.64)	66.69 (16.53)	61.55 (13.41)	66.64 (12.37)	<0.001
Sex					
Male	296 (60.7)	23 (54.8)	203 (62.5)	70 (57.9)	
Female	192 (39.3)	19 (45.2)	122 (37.5)	51 (42.1)	0.483
Year of Diagnosis (%)					0.007
2017	228 (46.7)	11 (26.2)	168 (51.7)	49 (40.5)	
2018	142 (29.1)	17 (40.5)	81 (24.9)	44 (36.4)	
2019	118 (24.2)	14 (33.3)	76 (23.4)	28 (23.1)	
Cancer Site (%)					0.067
Colon	349 (71.5)	38 (90.5)	226 (69.5)	85 (70.2)	
Rectosigmoid	16 (3.3)	<10	10 (3.1)	<10	
Rectum	123 (25.2)	<10	89 (27.4)	<10	
Metastatic Sites (%)					
Hepatic	341 (69.9)	21 (50.0)	247 (76.0)	73 (60.3)	<0.001
Pulmonary	137 (28.1)	<10	88 (27.1)	<10	0.277
Lymph Nodes	124 (25.4)	11 (26.2)	77 (23.7)	36 (29.8)	0.423
Adrenals	17 (3.5)	<10	11 (3.4)	<10	0.36
Peritoneum	129 (26.4)	21 (50.0)	79 (24.3)	29 (24.0)	0.001
Pleura	<10	<10	<10	<10	0.054
Osseous	24 (4.9)	<10	16 (4.9)	<10	0.68
Brain	<10	<10	<10	<10	0.823
Bone Marrow	<10	<10	<10	<10	0.101
Skin	<10	<10	<10	<10	0.778
Other	44 (9.0)	<10	23 (7.1)	<10	0.1

<10: data censored due to small cell size. *BRAF* = v-raf murine sarcoma viral oncogene homolog B1; SD = standard deviation.

**Table 2:** Treatment regimens for first- and second-line therapy among metastatic colorectal cancer patients.

Treatment Group	Overall	Mutant	Wild Type	Not Tested	*p*
	*n* = 488	*n* = 42	*n* = 325	*n* = 121	
First line (%)	327 (67.0)	24 (57.1)	243 (74.8)	60 (49.6)	
Capecitabine	75 (22.9)	<10	50 (20.6)	<10	0.325
CAPOX or FOLFOX	119 (36.4)	10 (41.7)	86 (35.4)	23 (38.3)	
CAPOX or FOLFOX + MAB	26 (8.0)	<10	23 (9.5)	<10	
FOLFIRI	48 (14.7)	<10	39 (16.0)	<10	
FOLFIRI + MAB	35 (10.7)	<10	28 (11.5)	<10	
Other	24 (7.3)	<10	17 (7.0)	<10	
Second line (%)	194 (39.8)	15 (35.8)	163 (50.2)	16 (13.2)	
Capecitabine	<10	<10	<10	<10	0.075
CAPOX or FOLFOX	54 (11.0)	<10	40 (12.3)	10 (8.3)	
CAPOX or FOLFOX + MAB	<10	<10	<10	<10	
FOLFIRI	49 (10.0)	<10	44 (13.5)	<10	
FOLFIRI + MAB	25 (5.1)	<10	21 (6.5)	<10	
Other	39 (8.0)	<10	35 (10.8)	<10	

<10: data censored due to small cell size. CAPOX = Capecitabine (Xeloda) and Oxaliplatin (Eloxatin); FOLFIRI = Fluorouracil (5-Fluorouracil, Efudex, Fluoroplex) and Irinotecan and Leucovorin Calcium; FOLFOX = Fluorouracil (5-Fluorouracil, Efudex, Fluoroplex) and Leucovorin Calcium and Oxaliplatin (Eloxatin); MAB = monoclonal antibody.

**Table 3:** Treatment durations for first- through fourth-line of therapy among metastatic colorectal cancer patients.

Summary (Days)	Line of Therapy			
	First Line	Second Line	Third Line	Fourth Line
Minimum	11	3	14	9
1st Quartile	84.5	63	50.5	70.25
Median	168	101.5	102.5	98
3rd Quartile	244.5	196	190.8	152.25
Maximum	1090	915	693	476

**Table 4:** Survival analyses from initiation of first-line therapy to all-cause death.

Strata	Median Survival in Months (95% CI)	1-Year Survival Probability (95% CI)	2-Year Survival Probability (95% CI)
Overall	20.01 (15.97–22.21)	0.66 (0.61–0.72)	0.38 (0.32–0.44)
*BRAF* status			
Wild type	20.03 (17.87–22.83)	0.71 (0.65–0.77)	0.39 (0.21–0.47)
Mutant	8.21 (6.54–12.62)	0.30 (0.16–0.56)	0.16 (0.06–0.43)
Not tested	21.00 (14.49-NA)	0.64 (0.52–0.79)	0.44 (0.30–0.62)
Treatment group			
Capecitabine	12.58 (10.02–20.99)	0.51 (0.41–0.64)	0.28 (0.19–0.42)
CAPOX or FOLFOX	27.04 (22.21–33.44)	0.77 (0.69–0.86)	0.58 (0.48–0.70)
CAPOX or FOLFOX + MAB	20.70 (15.41–32.13)	0.71 (0.55–0.92)	0.33 (18–0.60)
FOLFIRI	14.55 (11.43–22.70)	0.62 (0.49–0.79)	0.22 (0.12–0.42)
FOLFIRI + MAB	23.82 (21.19–34.27)	0.90 (0.80–1.00)	0.46 (0.30–0.70)
Other	10.15 (5.42–18.33)	0.36 (0.20–0.65)	0.16 (0.06–0.44)

*BRAF* = v-raf murine sarcoma viral oncogene homolog B1; CI = confidence interval; CAPOX = Capecitabine (Xeloda) and Oxaliplatin (Eloxatin); FOLFIRI = Fluorouracil (5-Fluorouracil, Efudex, Fluoroplex) and Irinotecan and Leucovorin Calcium; FOLFOX = Fluorouracil (5-Fluorouracil, Efudex, Fluoroplex) and Leucovorin Calcium and Oxaliplatin (Eloxatin); MAB = monoclonal antibody.

**Table 5:** Survival analyses from initiation of first-line therapy to cancer-specific death.

Strata	Median Survival in Months (95% CI)	1-Year Survival Probability (95% CI)	2-Year Survival Probability (95% CI)
Overall	21.19 (17.90–24.44)	0.69 (0.64–0.74)	0.43 (0.37–0.50)
*BRAF* status			
Wild type	22.08 (18.79–24.61)	0.72 (0.66–0.79)	0.43 (0.36–0.51)
Mutant	10.05 (6.54–14.39)	0.36 (0.20–0.63)	0.19 (0.07–0.48)
Not tested	31.24 (17.84–NA)	0.69 (0.57–0.83)	0.57 (0.43–0.74)
Treatment group			
Capecitabine	13.99 (10.12–22.10)	0.54 (0.44–0.68)	0.33 (0.23–0.48)
CAPOX or FOLFOX	29.04 (26.97–38.44)	0.80 (0.72–0.89)	0.65 (0.55–0.77)
CAPOX or FOLFOX + MAB	20.94 (15.41–NA)	0.74 (0.59–0.94)	0.40 (0.24–0.68)
FOLFIRI	15.05 (12.42–22.70)	0.64 (0.51–0.80)	0.23 (0.12–0.43)
FOLFIRI + MAB	24.44 (21.19–NA)	0.90 (0.80–1.00)	0.52 (0.35–0.76)
Other	10.45 (5.42–NA)	0.40 (0.23–0.70)	0.23 (0.10–0.53)

*BRAF* = v-raf murine sarcoma viral oncogene homolog B1; CI = confidence interval; CAPOX = Capecitabine (Xeloda) and Oxaliplatin (Eloxatin); FOLFIRI = Fluorouracil (5-Fluorouracil, Efudex, Fluoroplex) and Irinotecan and Leucovorin Calcium; FOLFOX = Fluorouracil (5-Fluorouracil, Efudex, Fluoroplex) and Leucovorin Calcium and Oxaliplatin (Eloxatin); MAB = monoclonal antibody; NA = not available.

## Data Availability

Individual-level data are not publicly available due to Canadian data privacy laws governing personal health information.

## Supplementary Materials

The following supporting information can be downloaded at: https://www.mdpi.com/article/10.3390/cancers15245748/s1. Figure S1: Kaplan–Meier curves depicting survival from time of initiation of second-line therapy to all-cause death. (A) Overall survival (B) stratified by *BRAF* status, (C) stratified by second-line therapy regimen. Table S1: Treatment regimens for third- and fourth-line therapy among metastatic colorectal cancer patients; Table S2: Survival analyses from initiation of second-line therapy to all-cause death; Table S3: Survival analyses from initiation of second-line therapy to cancer-specific death.
