# Supplementary material for: The Impact of BRAF Mutation Status on Survival Outcomes and Treatment Patterns among Metastatic Colorectal Cancer Patients in Alberta, Canada
Source: Cancers (Basel). 2023 Dec 8;15(24):5748. doi: 10.3390/cancers15245748 (PMC10741517; doi:10.3390/cancers15245748)
Supplement: Supplementary file 1 [file cancers-15-05748-s001.zip › cancers-2699752-supplementary.pdf]

Supplementary Materials: The Impact of *BRAF* Mutation Status on Survival Outcomes and Treatment Patterns among Metastatic Colorectal Cancer Patients in Alberta, Canada

R. Liam Sutherland, Devon J. Boyne, Darren R. Brenner and Winson Y. Cheung

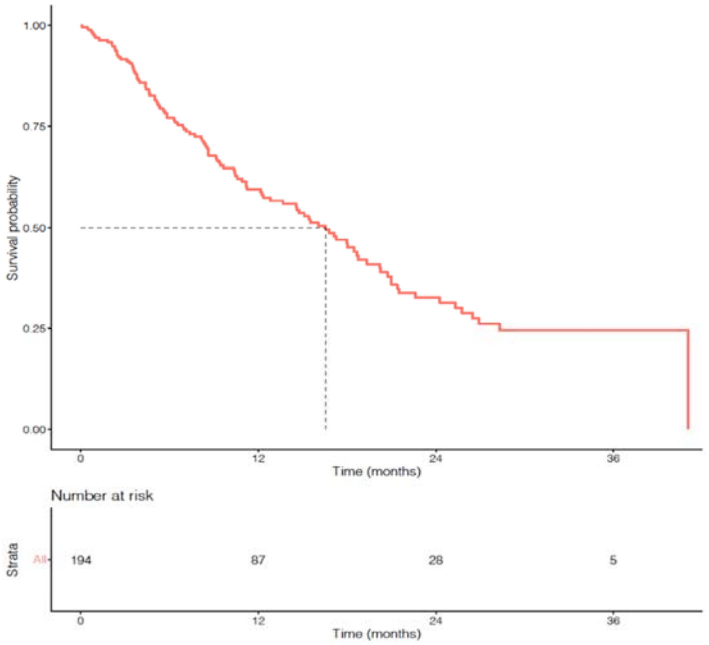

(**A**)

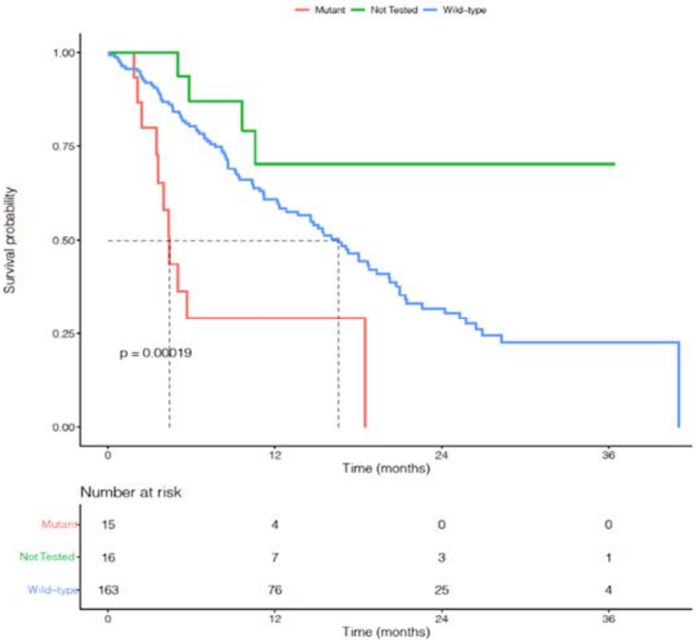

(**B**)

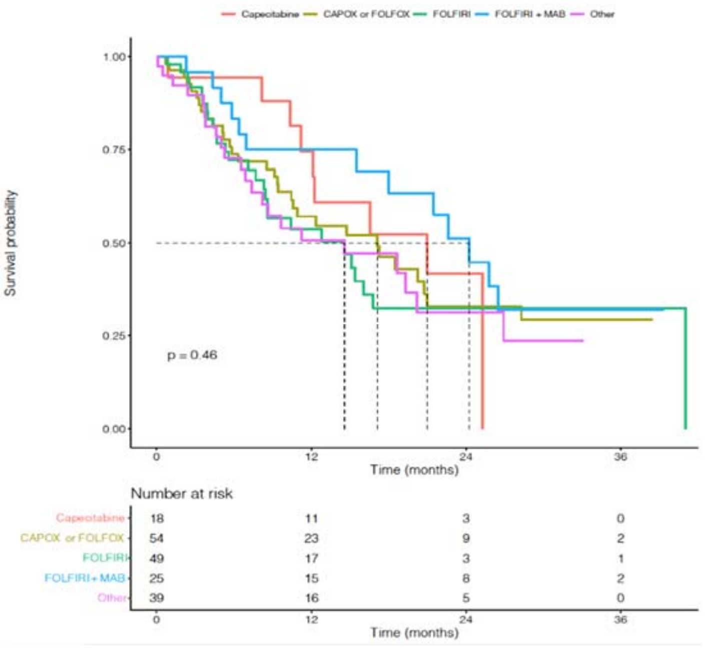

(**C**)

Figure S1: Kaplan-Meir curves depicting survival from time of initiation of second-line therapy to all-cause death. (A) Overall survival. (B) Stratified by *BRAF* status. (C) Stratified by second-line therapy regimen.

Table S1. Treatment regimens for third- and fourth-line therapy among metastatic colorectal cancer patients.

**Treatment Group**	**Overall**	**Mutant**	**Wild-Type**	**Not Tested**	**p**
	**n = 488**	**n = 42**	**n = 325**	**n = 121**	
Third-line (%)	96 (49.5)	<10	87 (53.4)	<10	
Capecitabine	<10	<10	<10	<10	0.892
CAPOX or FOLFOX	21 (21.9)	<10	20 (23.0)	<10	
CAPOX or FOLFOX + MAB	<10	<10	<10	<10	
FOLFIRI	12 (12.5)	<10	11 (12.6)	<10	
FOLFIRI + MAB	11 (11.5)	<10	11 (12.6)	<10	
Panitumumab	24 (25.0)	<10	22 (25.3)	<10	
Other	24 (25.0)	<10	19 (21.8)	<10	
Fourth-line (%)	30 (31.2)	<10	28 (32.2)	<10	
CAPOX or FOLFOX	<10	<10	<10	<10	0.92
CAPOX or FOLFOX + MAB	<10	<10	<10	<10	
FOLFIRI	<10	<10	<10	<10	
FOLFIRI + MAB	<10	<10	<10	<10	
Other	12 (40.0)	<10	10 (35.7)	<10	

<10: data censored due to small cell size.

CAPOX = Capecitabine (Xeloda) and Oxaliplatin (Eloxatin); FOLFIRI = Fluorouracil (5-Fluorouracil, Efudex, Fluoroplex) and Irinotecan and Leucovorin Calcium; FOLFOX = Fluorouracil (5-Fluorouracil, Efudex, Fluoroplex) and Leucovorin Calcium and Oxaliplatin (Eloxatin); MAB = monoclonal antibody.

Table S2. Survival analyses from initiation of second-line therapy to all-cause death.

Strata	Median survival in months (95% CI)	1 year survival probability (95% CI)	2 year survival probability (95% CI)
Overall	11.24 (9.36-14.75)	0.48 (0.40-0.56)	0.17 (0.12-0.26)
BRAF Status			
Wild type	12.16 (9.43-15.38)	0.50 (0.42-0.60)	0.16 (0.11-0.26)
Mutant	4.17 (3.48-18.46)	0.21 (0.08-0.58)	0.07 (0.01-0.47)
Not-tested	NA	0.51 (0.28-0.94)	0.51 (0.28-0.94)
Treatment Group			
Capecitabine	16.56 (11.17-NA)	0.68 (0.47-1.00)	0.31 (0.12-0.76)
CAPOX or FOLFOX	11.38 (9.36-18.46)	0.45 (0.33-0.62)	0.20 (10-0.38)
FOLFIRI	8.44 (5.58-15.11)	0.38 (0.25-0.59)	0.14 (0.06-0.37)
FOLFIRI + MAB	18.00 (6.96-26.48)	0.62 (0.45-0.87)	0.31 (0.15-0.63)
Other	8.64 (5.29--18.30)	0.40 (0.26-0.62)	0.10 (0.03-0.34)

BRAF = v-raf murine sarcoma viral oncogene homolog B1; CI = confidence interval; CAPOX = Capecitabine (Xeloda) and Oxaliplatin (Eloxatin); FOLFIRI = Fluorouracil (5-Fluorouracil, Efudex, Fluoroplex) and Irinotecan and Leucovorin Calcium; FOLFOX = Fluorouracil (5-Fluorouracil, Efudex, Fluoroplex) and Leucovorin Calcium and Oxaliplatin (Eloxatin); MAB = monoclonal antibody; NA = not available.

Table S3. Survival analyses from initiation of second-line therapy to cancer-specific death.

Strata	Median survival in months (95% CI)	1 year survival probability (95% CI)	2 year survival probability (95% CI)
Overall	12.35 (10.35-16.56)	0.52 (0.44-0.61)	0.22 (0.15-0.31)
BRAF Status			
Wild type	12.81 (10.45-16.79)	0.54 (0.46-0.63)	0.20 (0.14-0.31)
Mutant	4.37 (3.58-NA)	0.24 (0.09-0.63)	0.12 (0.02-0.65)
Not-tested	NA	0.61 (0.38-1.00)	0.61 (0.38-1.00
Treatment Group			
Capecitabine	15.56 (11.17-NA)	0.68 (0.47-1.00)	0.31 (0.12-0.76)
CAPOX or FOLFOX	12.35 (9.36-20.73)	0.52 (0.40-0.69)	0.24 (0.13-0.46)
FOLFIRI	8.61 (7.13-15.41)	0.42 (0.28-0.62)	0.16 (0.06-0.40)
FOLFIRI + MAB	21.49 (15.51-NA)	0.70 (0.53-0.93)	0.41 (0.22-0.76)
Other	8.64 (6.54-20.20)	0.42 (0.27-0.64)	0.15 (0.05-0.48)

BRAF = v-raf murine sarcoma viral oncogene homolog B1; CI = confidence interval; CAPOX = Capecitabine (Xeloda) and Oxaliplatin (Eloxatin); FOLFIRI = Fluorouracil (5-Fluorouracil, Efudex, Fluoroplex) and Irinotecan and Leucovorin Calcium; FOLFOX = Fluorouracil (5-Fluorouracil, Efudex, Fluoroplex) and Leucovorin Calcium and Oxaliplatin (Eloxatin); MAB = monoclonal antibody; NA = not available.
